# Evaluation and Comparison of Ketoconazole and Alkaline Glutaraldehyde Effects on Candida albicans Adherence to Three Denture Base Materials: An In Vitro Study

**DOI:** 10.7759/cureus.89273

**Published:** 2025-08-03

**Authors:** Palakayala Balaswamy, Gade P Krishna, Gottumukkala Vineela, Deepthi Batthula, Rophica J Mitta, Shyni R Gorremutchu

**Affiliations:** 1 Department of Prosthodontics, Sibar Institute of Dental Sciences, Guntur, IND

**Keywords:** candida albicans, denture base resin, disinfectant, glutaraldehyde, ketoconazole

## Abstract

Introduction: This study aimed to evaluate and compare the effects of two antimicrobial agents, ketoconazole and alkaline glutaraldehyde, on *Candida albicans* (*C. albicans*) adherence to three denture base materials: conventional heat-cured polymethyl methacrylate (PMMA), flexible denture base material, and injectable denture base material. The objectives of this study were to assess the efficacy of ketoconazole in reducing *C. albicans* adherence, evaluate the effectiveness of alkaline glutaraldehyde in preventing microbial attachment, and compare the performance of both agents across the tested materials to identify optimal strategies for controlling fungal infections.

Materials and methods: This in vitro study was conducted at the Sibar Institute of Dental Sciences, Guntur, India, between June 2023 and June 2024. Sixty uniform wax patterns (20 × 2 mm) were fabricated and divided into three groups (n = 20 each): Group 1 (conventional heat-cured PMMA, Dental Products of India (DPI®), Mumbai, India), Group 2 (flexible denture base material, Proflex®, Prevest DenPro, Jammu, India), and Group 3 (injectable denture base material, SR Ivocap® High Impact, Ivoclar Vivadent, Schaan, Liechtenstein). Each group was subdivided into subgroups A (ketoconazole, Nizral® 2%, Johnson & Johnson, New Brunswick, NJ, USA) and B (alkaline glutaraldehyde, Hospal®-G Plus, Septodont, Saint-Maur-des-Fossés, France). The samples were inoculated with *C. albicans* (ATCC 14053), treated with disinfectants for 10 minutes, and incubated on Sabouraud dextrose agar at 37°C for 24 and 48 hours. Colony-forming units (CFU/mL) were counted, and data were analyzed using independent t-tests, one-way analysis of variance (ANOVA), and repeated-measures ANOVA (p < 0.05).

Results: Pretreatment results indicated no significant intergroup differences (p > 0.05) in CFU/mL. At 24 hours, alkaline glutaraldehyde significantly reduced *C. albicans* counts compared with ketoconazole (p = 0.001), particularly in the injectable group. By 48 hours, both agents achieved significant reductions (p = 0.001), with alkaline glutaraldehyde maintaining superior efficacy, especially for injectable materials.

Conclusion: Both ketoconazole and alkaline glutaraldehyde effectively reduced *C. albicans* adherence, but alkaline glutaraldehyde demonstrated a faster and more potent antimicrobial action, particularly on injectable denture materials, making it suitable for rapid disinfection. Ketoconazole showed gradual and sustained efficacy, which is ideal for long-term fungal control.

## Introduction

The human oral cavity is a complex ecosystem that harbors a diverse array of microorganisms, among which *Candida albicans* (*C. albicans*) is of significant concern in dental practice. As an opportunistic pathogen, *C. albicans *exists as a commensal pathogen in the oral environment and colonizes surfaces such as the tongue, palate, teeth, and denture materials [1]. Its ability to adhere to these surfaces is a critical factor for survival and pathogenicity. Under favorable conditions, such as systemic immune suppression, alterations in the local oral environment, or the presence of virulence factors, *C. albicans *can transition from a benign commensal to a pathogenic state, leading to infections such as denture stomatitis [2]. This condition is particularly prevalent among denture wearers, manifesting in various forms depending on its severity and extent, and poses significant clinical challenges.

Denture stomatitis, a common clinical manifestation of *Candida *infection, affects individuals with complete or partial removable dentures. It is classified into three types based on its presentation: type I, characterized by localized pinpoint hyperemia; type II, marked by erythematous areas covering part or all of the denture-contacting mucosa; and type III, a combination of types I and II with additional granular inflammatory hyperplasia, typically affecting the midline of the hard palate and alveolar ridges [3]. Among the numerous etiological and predisposing factors, *Candida* species, particularly* C. albicans*, are the primary contributors to the initiation and progression of denture stomatitis [2,4]. In particular, the intaglio surface of maxillary dentures serves as a reservoir for these pathogens, facilitating their adherence and biofilm formation.

The adherence of* C. albicans* to denture base materials is a pivotal step in biofilm development and is influenced by the physical and chemical properties of the material, such as surface roughness, porosity, hydrophobicity, and surface free energy [5,6]. These properties are determined by the type of denture base material, whether acrylic, metal, or composite, and the fabrication techniques, including polymerization methods, surface modifications, and the incorporation of coatings and fibers [7,8]. The denture surface provides a favorable niche for microbial growth, exacerbating the risk of *C. albicans* colonization, particularly in conditions of poor oral and denture hygiene, ill-fitting prostheses, or failure to remove dentures at night [2]. Moreover, repeated inhalation or ingestion of microorganisms adhering to the mucosa or denture surfaces can pose additional risks, particularly in immunocompromised patients or those undergoing medical treatment, potentially leading to systemic infection [9].

Given the significant role of *C. albicans* in denture-related infections, the use of antimicrobial agents has emerged as a critical strategy for mitigating microbial adherence and biofilm formation in the oral cavity. These agents aim to reduce the risk of infection by targeting *C. albicans* on denture surfaces, thereby improving oral health outcomes for denture wearers [10]. However, the efficacy of antimicrobial agents can vary depending on the denture base material and surface characteristics [11]. This study aimed to evaluate and compare the effects of two antimicrobial agents, ketoconazole and alkaline glutaraldehyde, on the adherence of *C. albicans* to three different denture base materials. The purpose of this study was to investigate how these agents influence microbial colonization on denture surfaces, with the goal of identifying effective strategies to mitigate fungal infections, such as denture stomatitis. Specifically, this study aimed to assess the efficacy of ketoconazole in reducing *C. albicans* adherence to three denture base materials, evaluate the effectiveness of alkaline glutaraldehyde in preventing microbial attachment to the same materials, and compare the performance of both antimicrobial agents to determine which was more effective in minimizing *C. albicans* adhesion on the tested denture base materials.

## Materials and methods

This in vitro study was conducted at the Department of Prosthodontics, Sibar Institute of Dental Sciences, Guntur, India, from June 2023 to June 2024. Ethical approval was not required as the study was conducted in vitro and did not involve human or animal subjects.

The sample size was determined using G*Power software, version 3.1.9.2 (Heinrich-Heine-Universität Düsseldorf, Düsseldorf, Germany), based on a previous study comparing *Candida* colonies (CFU/mL) in injectable and heat-cured denture base materials, with an effect size of 0.55 [12]. The minimum required sample size was calculated as 60 (20 per group), with a power of 90% and an alpha error of 5%. This ensured adequate statistical power to detect significant differences between the different denture materials in terms of their antimicrobial effects.

These samples were equally divided into three groups of 20, each corresponding to one of the denture base materials: Group 1 (n = 20) consisted of conventional heat-cured polymethyl methacrylate (PMMA) (DPI®, Dental Products of India, Mumbai, India), Group 2 consisted of flexible denture base material (Proflex®, Prevest DenPro, Jammu, India), and Group 3 consisted of injectable denture base material (SR Ivocap® High Impact, Ivoclar Vivadent, Schaan, Liechtenstein). Each group was further subdivided into two subgroups of 10 samples each, based on the antimicrobial agent used: Subgroup A with immersion in ketoconazole (Nizral® 2%, Johnson & Johnson, New Brunswick, NJ, USA) and Subgroup B with immersion in alkaline glutaraldehyde (Hospal®-G Plus, Septodont, Saint-Maur-des-Fossés, France).

The methodology began with mold fabrication using a standard aluminium block (100 × 30 × 2 mm), where a computerized milling machine created a mold space of 20 × 2 mm to fabricate 60 uniform wax patterns. The modelling wax (Hindustan Modelling Wax No. 2 HDP, Hindustan Dental Products, Hyderabad, India) was melted and poured into the mold space, allowed to set, and carefully removed to produce 60 polished wax patterns, which were divided into three groups of 20 for processing with the respective denture base materials. In Group 1 (conventional heat-cured PMMA), wax patterns were flasked using a two-pour technique in dental flasks (Jabbar and Company, Mumbai, India). The type III gypsum product (Goldstone® Dental Stone Type III, Asian Chemicals, Rajkot, India) was mixed according to the manufacturer’s instructions and poured into the flask base to form the first pour, immersing the 20 wax patterns until visible from the top. After setting, a cold mold seal (DPI® Cold Mould Seal, Dental Products of India, Mumbai, India) was applied uniformly, followed by a second pour of dental stone until the second rim of the flask was filled. The flask was closed, clamped, and the excess material was flushed out, allowing it to set for 30 minutes. The dewaxing unit (Unident Instruments India Pvt. Ltd., New Delhi, India) was set to 100°C, and the flask was immersed for five minutes to remove wax using a hot water flush. After ensuring that no residual wax remained and that the flask was dried, a cold mold seal was reapplied. The heat-cured PMMA (DPI, Mumbai, India) polymer and monomer were mixed in a porcelain jar according to the manufacturer’s instructions, packed into the mold space at dough consistency, and placed under a hydraulic bench press (Unident Instruments India Pvt. Ltd., New Delhi, India) at 100 pounds per square inch (PSI). Excess material was removed and the flasks were bench cured for 30 minutes, followed by curing in an acrylizer (Unident Instruments India Pvt. Ltd., New Delhi, India) at 90°C for 90 minutes. After overnight cooling, deflasking was performed, and the samples were trimmed and smoothed using sandpaper grits of sandpaper (Sand Master® NXT, Abrasive Technologies, Chennai, India), ensuring uniform thickness.

For Group 2 (flexible denture base material), wax patterns were placed in specialized flasks for flexible denture fabrication using the two-pour technique with type III dental stone. Before the first pour set, spruing wax runner bars were attached to the wax patterns to facilitate material flow. After applying a cold mold seal and completing the second pour, the flask was clamped for 30 minutes. Dewaxing was performed at 100°C for five minutes, followed by a hot water flush to remove the wax, and the flask was dried. A Proflex® cartridge was sprayed with silicone to prevent adhesion, plasticized in an electric cartridge furnace at 550-560°F for 15-20 minutes, and compressed into the flask at 100 PSI for 3-5 minutes. After bench cooling for 15-20 minutes, deflasking was performed, and the samples were trimmed and smoothed with sandpaper to ensure uniform thickness.

In Group 3 (injectable denture base material), wax patterns were flasked in specialized flasks for denture fabrication using the two-pour technique with type III dental stone. Spruing wax runner bars were attached prior to the first pour set. After applying a cold mold seal and completing the second pour, the flask was clamped for 30 minutes. Dewaxing was performed at 100°C for five minutes, followed by hot water flushing and drying. An Ivocap® funnel was positioned in the flask, and the SR Ivocap® High Impact monomer was poured into a capsule containing polymer, mixed for five minutes in a Cap Vibrator, and injected into the mold space via the sprue method. The flask was placed in a water bath (Unident Instruments India Pvt. Ltd., New Delhi, India) at 74-100°C for curing. After deflasking, the samples were trimmed and smoothed with sandpaper to ensure a uniform thickness.

**Figure 1:**
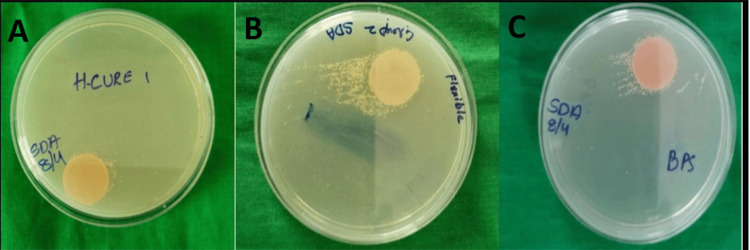
Pretreatment Candida colony formation on Sabouraud dextrose agar plates (A) Conventional heat-cured polymethyl methacrylate (PMMA). (B) Flexible denture base material. (C) Injectable denture base material. Original images of samples from the study

*C. albicans* (ATCC 14053) and Sabouraud dextrose agar (SDA) (Himedia, Mumbai, India) were obtained from the Department of Microbiology. A *C. albicans* suspension (1:10 dilution) was prepared by mixing the strain with physiological saline solution and standardized using a McFarland test tube (BioMerieux Inc., Marcy-l’Étoile, France). *C. albicans* growth was microscopically confirmed. Denture base samples were sterilized under UV radiation, dried, and immersed in *Candida* suspension for one minute. They were then inoculated on SDA in Petri dishes and incubated at 37°C for 24 hours in a bacteriological incubator (Universal®, Labindia Instruments, Mumbai, India). The initial colony-forming units (CFU) were visually counted (Figure 1).

**Figure 2:**
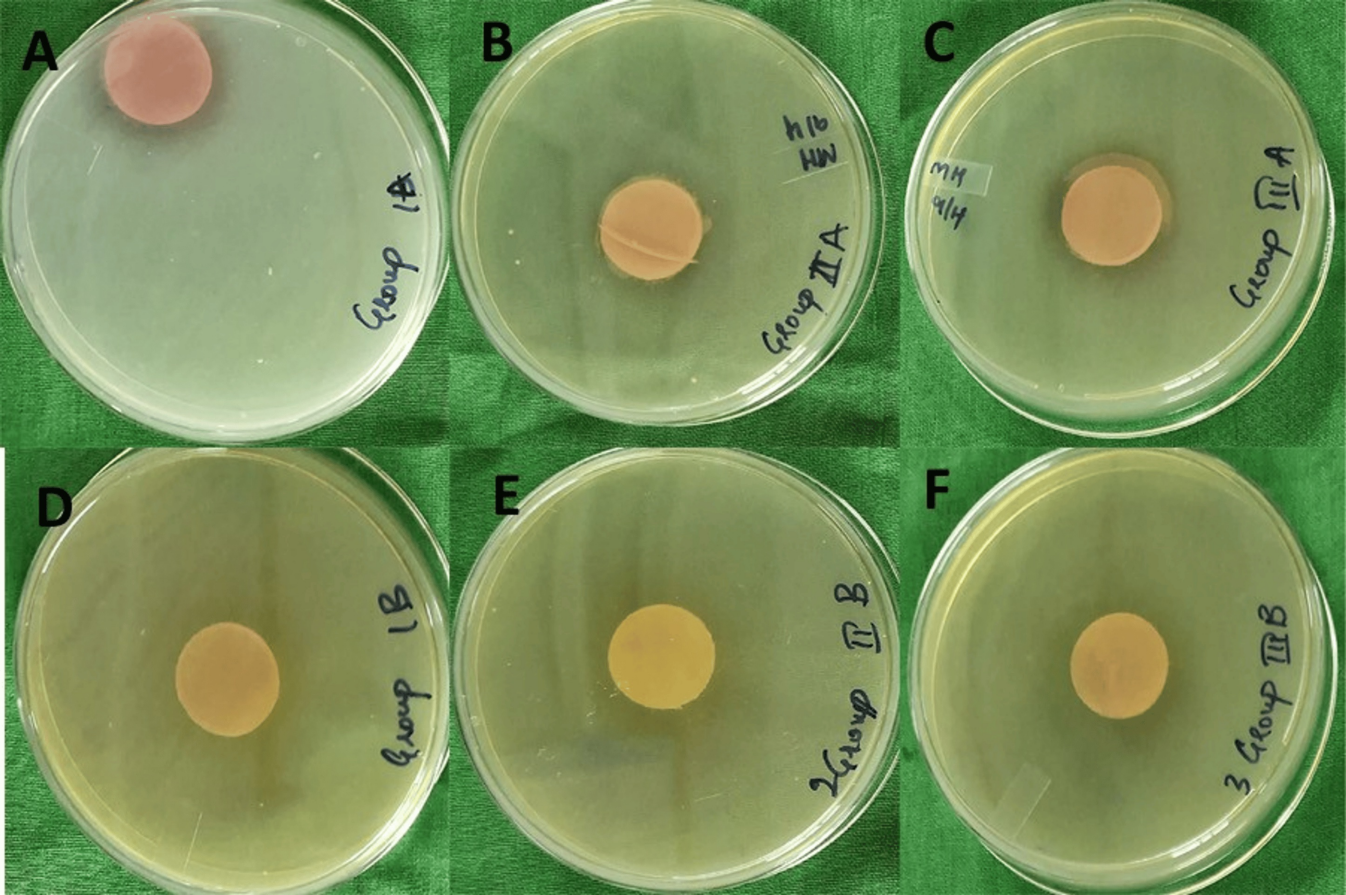
Candida colonies after immersion in disinfectant on Sabouraud dextrose agar plates for different groups (A) Conventional heat-cured polymethyl methacrylate (PMMA) group immersed in ketoconazole. (B) Flexible denture base material group immersed in ketoconazole. (C) Injectable denture base material group immersed in ketoconazole. (D) Conventional heat-cured PMMA group immersed in alkaline glutaraldehyde. (E) Flexible denture material group immersed in alkaline glutaraldehyde. (F) Injectable denture material group immersed in alkaline glutaraldehyde Original images of samples from the study

For disinfection, samples from each group were divided into Subgroup A (immersed in ketoconazole for 10 minutes) and Subgroup B (immersed in alkaline glutaraldehyde for 10 minutes). The 10-minute immersion time was chosen as it balances effectiveness with material safety, avoiding damage to denture surfaces such as PMMA [7]. It aligns with standardized protocols for antifungal and disinfectant agents, ensuring clinical practicality and biofilm penetration [10]. Treated samples were inoculated on SDA, incubated for 24 and 48 hours, and the final CFU were counted and statistically analyzed (Figure 2).

To ensure calibration and reliability, all equipment was calibrated before use according to manufacturer specifications, and measurements, such as CFU, were performed by two independent observers to ensure consistency. Interobserver reliability was assessed using Cohen’s kappa coefficient, achieving a value of 0.85, indicating high agreement.

Statistical analysis

Data were analyzed using IBM SPSS Statistics for Windows, Version 20 (Released 2011; IBM Corp., Armonk, New York, United States). Data normality was confirmed using the Shapiro-Wilk test (p > 0.05) to ensure parametric test suitability. The study compared the antimicrobial efficacy of denture base materials treated with ketoconazole and alkaline glutaraldehyde using an independent t-test analysis between subgroups and one-way analysis of variance (ANOVA) for intergroup comparisons, with significance set at p < 0.05. The comparison of the antimicrobial effect of the denture base material at multiple time intervals was performed using repeated-measures ANOVA.

## Results

Pretreatment colony counts were similar across all subgroups, with no statistically significant difference (p > 0.05). At 24 hours, alkaline glutaraldehyde showed a significantly greater reduction (p = 0.001) in all groups than ketoconazole. After 48 hours, both agents further reduced colony counts; however, alkaline glutaraldehyde maintained superior efficacy (p = 0.001), particularly in the injectable group. Alkaline glutaraldehyde demonstrated rapid and stronger antimicrobial action within 24 hours, likely due to its biocidal mechanism, whereas ketoconazole exhibited gradual fungistatic suppression by 48 hours. Both treatments were effective long-term, but alkaline glutaraldehyde was more efficient for immediate disinfection (Table 1).

**Table 1:** Comparative analysis of antimicrobial efficacy in different groups between different treatment modalities *p < 0.05 denotes statistical significance using an independent t-test for intergroup comparison of colonies in colony-forming units/mL (CFU/mL). Colonies are represented in the form of mean and standard deviation, where n denotes the number of samples in each group

Groups	Subgroups	n	Pretreatment (CFU/mL × 10²)	t value	p-value	24 hours (CFU/mL × 10²)	t value	p-value	48 hours (CFU/mL × 10²)	t value	p-value
Conventional heat-cured polymethyl methacrylate	A (ketoconazole)	10	3.34 ± 0.85	0.95	0.350	1.45 ± 0.18	12.99	0.001*	0.26 ± 0.12	12.27	0.001*
	B (alkaline glutaraldehyde)	10	3.67 ± 0.68			0.25 ± 0.23			0.22 ± 0.13		
Flexible denture base material	A (ketoconazole)	10	3.32 ± 0.54	0.43	0.665	2.34 ± 0.43	14.08	0.001*	0.34 ± 0.12	20.12	0.001*
	B (alkaline glutaraldehyde)	10	3.18 ± 0.85			0.28 ± 0.12			0.26 ± 0.12		
Injectable denture base material	A (ketoconazole)	10	3.34 ± 0.87	0.24	0.811	1.24 ± 0.42	7.27	0.001*	0.28 ± 0.13	14.19	0.001
	B (alkaline glutaraldehyde)	10	3.26 ± 0.58			0.16 ± 0.21			0.11 ± 0.12		

Pretreatment colony counts showed no significant differences between the groups for either treatment (ketoconazole, p = 0.997; alkaline glutaraldehyde, p = 0.273). At 24 hours, ketoconazole exhibited marginally higher variability (p = 0.068), with injectable denture material showing the lowest number of colonies. In contrast, glutaraldehyde maintained consistent efficacy across all groups (p = 0.492), with the lowest counts observed in the injectable denture material. After 48 hours, both treatments achieved further reduction; however, no significant intergroup differences were observed (ketoconazole, p = 0.335; glutaraldehyde, p = 0.636). Glutaraldehyde provided uniform and rapid antimicrobial action across all groups, whereas ketoconazole efficacy varied slightly, particularly in flexible materials. The lack of significant differences suggests that both treatments eventually achieved comparable *Candida* suppression, although glutaraldehyde acted faster (Table 2).

**Table 2:** Comparative analysis of antimicrobial efficacy between different groups for different treatment modalities p > 0.05 denotes no statistical significance using one-way analysis of variance (ANOVA) test for intergroup comparison of colonies in colony-forming units/mL (CFU/mL). Colonies are represented in the form of mean and standard deviation

Disinfectant used	Groups	Pretreatment (CFU/mL × 10²)	F value	p-value	24 hours (CFU/mL × 10²)	F value	p-value	48 hours (CFU/mL × 10²)	F value	p-value
A (ketoconazole)	Conventional heat-cured polymethyl methacrylate	3.34 ± 0.85	0.002	0.997	1.45 ± 0.18	3.86	0.068	0.26 ± 0.12	1.13	0.335
	Flexible denture base material	3.32 ± 0.54			2.34 ± 0.43			0.34 ± 0.12		
	Injectable denture base material	3.34 ± 0.87			1.24 ± 0.42			0.28 ± 0.13		
B (alkaline glutaraldehyde)	Conventional heat-cured polymethyl methacrylate	3.67 ± 0.68	1.36	0.273	0.25 ± 0.23	0.72	0.492	0.22 ± 0.13	0.45	0.636
	Flexible denture base material	3.18 ± 0.85			0.28 ± 0.12			0.26 ± 0.12		
	Injectable denture base material	3.26 ± 0.58			0.16 ± 0.21			0.11 ± 0.12		

Comparative analysis of antimicrobial efficacy across multiple time intervals revealed highly significant reductions (p = 0.001) in *Candida* colony counts for both ketoconazole (Subgroup A) and alkaline glutaraldehyde (Subgroup B) treatments across all groups. In the conventional heat-cured PMMA group, alkaline glutaraldehyde demonstrated superior immediate efficacy compared with ketoconazole at 24 hours. This pattern persisted in flexible denture base materials, where ketoconazole showed a slower action than alkaline glutaraldehyde. The injectable denture base material group exhibited the most dramatic reduction with glutaraldehyde, achieving near-complete eradication within 48 hours. Although both treatments showed statistically significant time-dependent efficacy (p = 0.001), alkaline glutaraldehyde consistently demonstrated faster and more pronounced antimicrobial effects across all time points and material types. These findings suggest that while both agents are effective for *Candida* control, alkaline glutaraldehyde's rapid biocidal action makes it particularly suitable for immediate disinfection applications, whereas the gradual fungistatic effect of ketoconazole may be more appropriate for sustained therapeutic use (Table 3).

**Table 3:** Comparative analysis of antimicrobial efficacy at multiple time intervals *p < 0.05 denotes statistical significance using repeated-measures analysis of variance (ANOVA) test for intergroup comparison of colonies in colony-forming units/mL (CFU/mL) at multiple time intervals. Colonies are represented in form of mean and standard deviation, where n denotes number of samples in each group

Groups	Subgroups	n	Pretreatment (CFU/mL × 10²)	24 hours (CFU/mL × 10²)	48 hours (CFU/mL × 10²)	F value	p-value
Conventional heat-cured polymethyl methacrylate	A (Ketoconazole)	10	3.34 ± 0.85	1.45 ± 0.18	0.26 ± 0.12	94.07	0.001*
	B (Alkaline glutaraldehyde)	10	3.67 ± 0.68	0.25 ± 0.23	0.22 ± 0.13	221.71	0.001*
Flexible denture base material	A (Ketoconazole)	10	3.32 ± 0.54	2.34 ± 0.43	0.34 ± 0.12	140.97	0.001*
	B (Alkaline glutaraldehyde)	10	3.18 ± 0.85	0.28 ± 0.12	0.26 ± 0.12	112.71	0.001*
Injectable denture base material	A (Ketoconazole)	10	3.34 ± 0.87	1.24 ± 0.42	0.28 ± 0.13	77.32	0.001*
	B (Alkaline glutaraldehyde)	10	3.26 ± 0.58	0.16 ± 0.21	0.11 ± 0.12	247.34	0.001*

## Discussion

The results of this study indicated that both ketoconazole and alkaline glutaraldehyde significantly reduced *C. albicans* colonization across all denture base materials, with statistically significant reductions observed at 24 and 48 hours. However, alkaline glutaraldehyde consistently outperformed ketoconazole in terms of the speed and extent of microbial reduction, particularly at 24 hours posttreatment. Alkaline glutaraldehyde, a broad-spectrum biocide, disrupts microbial cell walls and proteins, leading to rapid cell death [13]. In contrast, ketoconazole, an antifungal agent, inhibits ergosterol synthesis in fungal cell membranes, exerting a fungistatic effect that requires longer exposure time for maximal efficacy [14]. These mechanistic differences explain why alkaline glutaraldehyde achieved near-complete eradication of* C. albicans* by 48 hours, especially in the injectable denture base material group, whereas ketoconazole showed a more gradual reduction in viability.

Shen et al. [15] reported that alkaline glutaraldehyde-based disinfectants did not cause significant surface alterations in conventional heat-cured PMMA after 12 hours of immersion; however, the flexural strength was significantly affected. Ferreira et al. [16] reported that the incorporation of antifungal agents into denture base materials significantly inhibited* C. albicans*, consistent with our observation of the fungistatic effect of ketoconazole. The superior immediate efficacy of alkaline glutaraldehyde in our study may be attributed to its ability to penetrate biofilms more effectively. Turecka et al. [17] found that ketoconazole alone was less effective as an antifungal agent against *C. albicans*.

The results of this study also revealed variations in antimicrobial efficacy based on the denture base material. The injectable denture base material exhibited the most significant reduction in colonies of *C. albicans* with alkaline glutaraldehyde, achieving near-complete eradication within 48 hours. This could be attributed to the smoother surface characteristics of the injectable materials, which may reduce microbial adhesion sites compared to conventional PMMA or flexible materials [18]. The current study demonstrated that flexible denture base materials exhibited marginally elevated colony counts relative to PMMA variations at 24 and 48 hours when subjected to ketoconazole and alkaline glutaraldehyde. This phenomenon may be attributed to the rougher surface characteristics of flexible dentures compared to those of traditional PMMA, as documented in a previous study by Singh et al. [19]. This assertion was further corroborated by a review conducted by Binaljadm [20], which indicated a greater degree of surface roughness in flexible dentures than in conventional PMMA dentures.

These findings are consistent with those of previous studies. For example, Chandra et al. [21] found that the surface roughness and hydrophobicity of denture materials significantly influenced *C. albicans* adhesion, with smoother surfaces, such as those of injectable materials, reducing microbial attachment. Conversely, Singh et al. [19] reported that flexible denture materials, owing to their elasticity and surface porosity, may retain more microbial biofilms, which could explain the slightly higher CFU/mL counts observed with ketoconazole in our flexible-material group. The lack of significant intergroup differences in pretreatment colony counts suggests that material-specific differences became more apparent after treatment with disinfectants, likely due to the interaction between the disinfectants and surface properties.

The superior performance of alkaline glutaraldehyde can be attributed to its biocidal mechanism, which rapidly disrupts microbial cell integrity, making it highly effective against *C. albicans* biofilms [13]. This is particularly relevant for denture surfaces, where biofilms are the primary cause of oral stomatitis. The rapid action of glutaraldehyde is advantageous in clinical settings that require immediate disinfection, such as denture maintenance. Ketoconazole’s slower, fungistatic action and effectiveness over 48 hours suggest that it may be better suited for sustained therapeutic applications, such as topical treatments for denture stomatitis [14].

Material-specific differences likely stem from surface characteristics. The current investigation demonstrated the lowest colony counts of *C. albicans* in injectable denture base substances formulated with alkaline glutaraldehyde, a finding that corroborates previous research [22]. Injection molding resins are supplied in preformulated cartridges, whereas in traditional methods, powder and liquid are manually combined, potentially resulting in the entrapment of air bubbles and heightened surface porosity. In contrast, Young et al. [23] indicated that the adherence of* C. albicans* to injection-molded acrylic materials was not significantly different from that of traditional pressure packs and self-cured acrylic resins. The discrepancy between the findings of Young et al. and the current study may be attributed to the application of sonication for the detachment of adhered *C. albicans* from the samples, as opposed to shaking, which could potentially result in detrimental effects on the cell wall of *C. albicans*.

Flexible materials, with their elastic and porous nature, may retain more microbes, as observed by the slightly higher variability with ketoconazole. Conventional PMMA, a widely used material, has a moderately rough surface, which may explain its intermediate performance [18]. The lack of significant intergroup differences suggests that both disinfectants eventually achieve comparable *C. albicans *suppression; however, the speed of action varies, with glutaraldehyde being faster acting.

Clinical implications

These findings have significant clinical implications in the management of denture-related infections. The rapid and potent antimicrobial action of alkaline glutaraldehyde makes it an ideal choice for chairside disinfection of dentures, particularly for injectable materials, which are increasingly used in modern prosthodontics. Its ability to achieve near-complete *C. albicans* eradication within 24 hours suggests that it may reduce the risk of denture stomatitis in clinical practice. Ketoconazole, while slower, remains effective for long-term management, particularly as a topical agent in patients with recurrent fungal infections. Clinicians should consider material-specific properties when selecting disinfectants; for instance, injectable denture materials may benefit most from glutaraldehyde-based protocols, whereas flexible materials may require extended ketoconazole exposure to overcome surface-related microbial retention.

Limitations

This study had several limitations. First, the in vitro design limits the direct extrapolation to clinical settings, where saliva, oral microbiota, and patient factors (such as immune status) influence *C. albicans* adhesion and biofilm formation. Second, this study used a single *C. albicans* strain, which may not represent the diversity of clinical isolates, some of which may exhibit antifungal resistance. Third, the 10-minute immersion time for disinfectants may not reflect real-world protocols, where longer or repeated exposures are common. Finally, the study did not assess long-term material properties such as surface degradation or color stability, which could be affected by repeated disinfectant use. Conversely, considering the significant influence of surface roughness on microbial adhesion, it is strongly advised to evaluate this parameter using an electronic microscope. Future studies should incorporate in vivo models, multiple *C. albicans* strains, and extended exposure times to better simulate the clinical conditions.

## Conclusions

This study demonstrated that both ketoconazole and alkaline glutaraldehyde effectively reduced *C. albicans* adhesion to denture base materials, with alkaline glutaraldehyde showing superior immediate efficacy, particularly on injectable denture materials. These findings highlight the importance of selecting disinfectants based on their mechanisms and the surface properties of the denture materials. Although both agents are viable for managing denture-related infections, alkaline glutaraldehyde is better suited for rapid disinfection, and ketoconazole may be preferred for sustained antifungal therapy. Further research is needed to validate these findings in clinical settings and explore long-term material compatibility.
